# Substance P Depolarizes Lamprey Spinal Cord Neurons by Inhibiting Background Potassium Channels

**DOI:** 10.1371/journal.pone.0133136

**Published:** 2015-07-21

**Authors:** Carolina Thörn Pérez, Russell H. Hill, Sten Grillner

**Affiliations:** Nobel Institute for Neurophysiology, Department of Neuroscience, Karolinska Institutet, Stockholm, Sweden; University of Houston, UNITED STATES

## Abstract

Substance P is endogenously released in the adult lamprey spinal cord and accelerates the burst frequency of fictive locomotion. This is achieved by multiple effects on interneurons and motoneurons, including an attenuation of calcium currents, potentiation of NMDA currents and reduction of the reciprocal inhibition. While substance P also depolarizes spinal cord neurons, the underlying mechanism has not been resolved. Here we show that effects of substance P on background K^+^ channels are the main source for this depolarization. Hyperpolarizing steps induced inward currents during whole-cell voltage clamp that were reduced by substance P. These background K^+^ channels are pH sensitive and are selectively blocked by anandamide and AVE1231. These blockers counteracted the effect of substance P on these channels and the resting membrane potential depolarization in spinal cord neurons. Thus, we have shown now that substance P inhibits background K^+^ channels that in turn induce depolarization, which is likely to contribute to the frequency increase observed with substance P during fictive locomotion.

## Introduction

Background channels play a fundamental role in determining the neuronal resting membrane potential, input resistance and excitability [1]. Background K^+^ channels (previously called leak channels) can be regulated by voltage-independent factors as pH and temperature, second messengers [2] and are common targets for neuromodulation [3, 4]. Inhibition of background K^+^ channels induces membrane depolarization, increased membrane resistance and consequently increases the firing rate, which could have great impact at the cellular level as well as on the network activity.

The modulatory effect of substance P on the neuronal network underlying locomotion has been studied in the spinal cord [5, 6]. In mammals, bath application of substance P during fictive walking increases the locomotor frequency [6]. In the lamprey spinal cord, substance P is released endogenously during fictive swimming [7] and it increases the burst frequency [8]. The cellular mechanisms underlying the increase in burst frequency include a membrane depolarization and a potentiation of NMDA current [8, 9], as well as a reduction of the crossed inhibition, via endocannabinoids [10]. The membrane depolarization is accompanied by an increased input resistance at resting membrane potential suggesting that this effect may be mediated by a decrease in outward background K^+^ conductance. Our goal here is to examine the possible influence of substance P on background K^+^ channels, which contribute to setting the resting membrane potential and which may modulate the excitability of central pattern generator (CPG) neurons.

The two-pore potassium channels TREK-1, TREK-2 and TRAAK regulate cellular excitability by providing temperature-dependent leak of potassium [11]. TASK-1 and TASK-2 also belong to the two-pore K^+^ channel family and are characterized by their pH sensitivity [12]. When TASK-1 channels open they allow diffusion of K^+^ ions across the membrane and show a small rectification [13]. G-protein coupled receptors have been reported to inhibit TASK-1 currents [3]. Substance P binds to NK_1_ receptors which are G protein-coupled and known to induce the activation of phospholipase C and produce inositol triphosphate [14].

Modulation of K^+^ channels by activation of metabotropic receptors has been studied previously in lamprey neurons. mGluR1, but not mGluR5 mediates depolarization by blocking K^+^ currents [15]. In mammals, 5-HT and substance P have been shown to inhibit background K^+^ channels of the two-pore, TASK-1 subtype [3] [16] and substance P modulation of the TASK-1 subtype has also been implicated in regulating the respiratory rhythm generation [4].

Our results indicate that substance P inhibits a K^+^ conductance by interacting with background K^+^ channels. We further present evidence these channels likely belong to the two-pore, TASK-1 K^+^ channel subtype based on their unique pH sensitivity and the selective blockade by anandamide and AVE1231.

## Materials and Methods

Experiments were performed on the isolated spinal cords of young adult sea lampreys (*Petromyzon marinus*) caught from the wild in Massachusetts (USA) in accordance and approval with field and health guidelines (Acme Lamprey Company of Harrison, State of Maine) and transported to Sweden by authorized agents for ACME Lamprey Company. The research project was carried out in strict accordance with the recommendations in the institutional guidelines of The Animal Research Ethical Committee, Stockholm. The protocol was approved by Stockholm Norra djurförsöksetiska nämnd (N113/12). All surgery was performed under MS-222 anesthesia, and all efforts were made to minimize suffering.

### Spinal cord preparation

**Fig 1:**
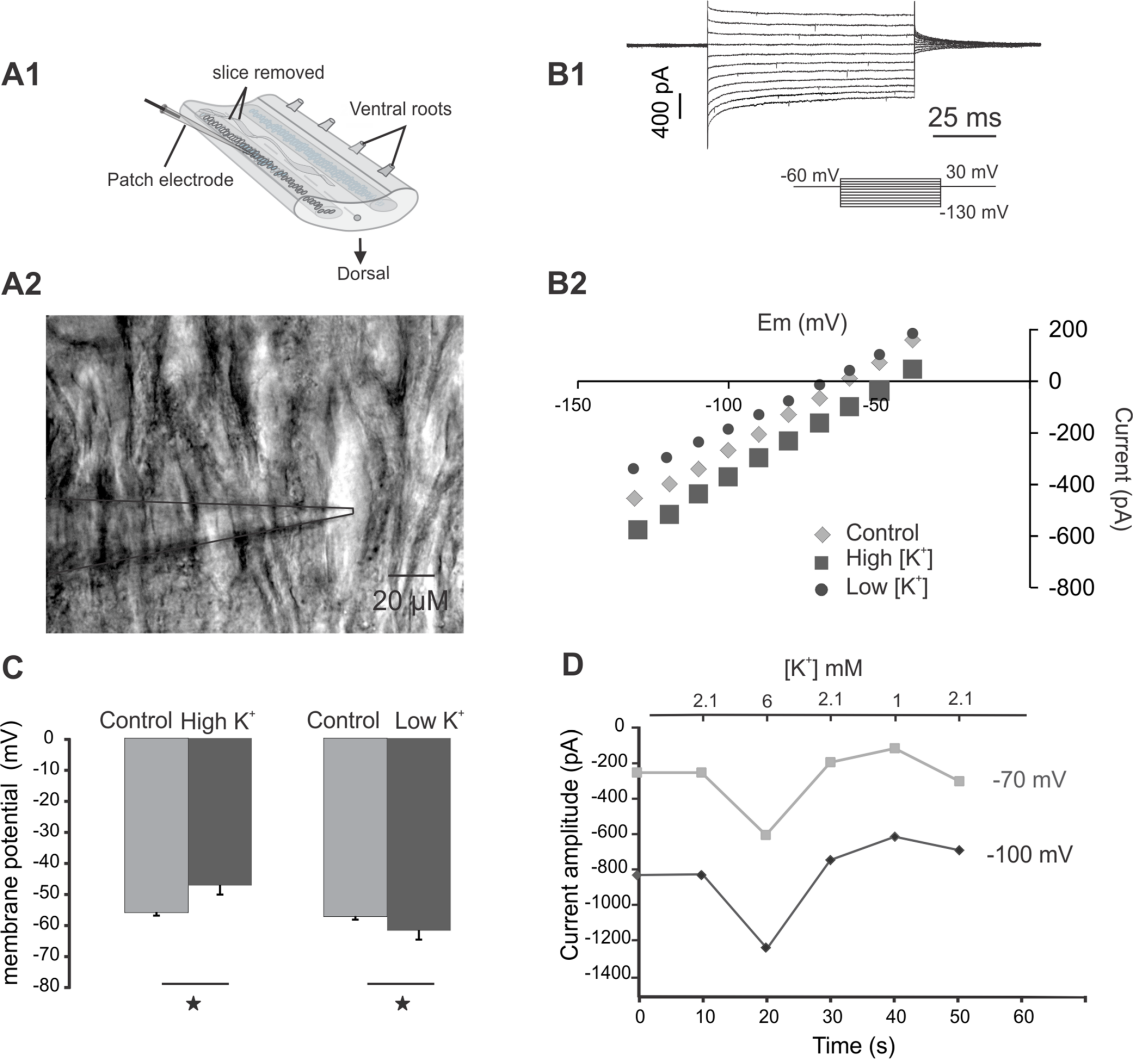
A1. Experimental arrangement of the isolated spinal cord. A2.Photomicrograph of a patched neuron exposed by slicing the spinal cord on the ventral side. The micropipette is shown to the left of the cell. B1. Current traces from voltage steps (-30 to -130) applied to a spinal neuron in the presence of TTX (1 μM) and Kynurenic acid (2 mM). B2. The current-voltage (I-V) relationship with reversal potential at ~60 mV at control conditions, ~50 mV with high extracellular K^+^ and ~64 mV with low K^+^ (Control: 2.1 mM, Low: 1 mM and high: 6 mM). C. Membrane potential changes with different extracellular concentrations of K^+^ during current clamp recording (Control: 2.1 mM, Low: 1 mM and high: 6 mM, single star = p < 0.05, n = 3). D. Current measurements from hyperpolarizing voltage steps (-70 and -100 mV) under voltage clamp during application of different extracellular K^+^ concentrations (2.1, 6 and 1 mM). Holding potential was -60 mV.

Animals were anesthetized with tricaine methanesulfonate (MS 222, 100 mg/l; Sigma–Aldrich, Sweden), decapitated, and the spinal cord was dissected and kept at 4–8°C in a saline solution of the following composition (in mM): 137.9 NaCl, 2.1 KCl, 2.6 CaCl_2_, 1.8 MgCl_2_, 4 glucose, 5 HEPES. The pH was adjusted to 7.4 with 1 M NaOH. The osmolarity was adjusted to 270 mOsm with distilled water. The spinal cord and musculature of approximately 8 segments were pinned down to a Sylgard chamber. The protective meninx primitiva was removed and the spinal cords were isolated and placed in a cooled microslicer with the ventral side up. A horizontal layer of about 40 μm above the gray matter was removed from the ventral surface to facilitate visibility and penetration of the patch electrode into the tissue (Fig 1A). The spinal cords were then pinned to a cooled sylgard-lined chamber. Solutions of pharmacological agents were bath-applied at a perfusion rate of 1 ml/min into a chamber volume of 1ml.

### Pharmacology

For the characterization of potassium currents, the spinal cords were incubated in Kynurenic acid (2 mM) and TTX (1 μM) (Sigma/Aldrich) to block glutamate receptors and sodium channels. Substance P (Sigma-Aldrich) was dissolved in water with 0.05 M acetic acid to prevent oxidation and 1% bovine serum albumin to increase the solution stability. Frozen 1 mM aliquots were stored at –20°C. They were dissolved in physiological solution to reach 1 μM concentration of substance P and applied for 20 minutes. Acetic acid alone at this final concentration had no effect on the pH or background K^+^ channels. Modified concentrations of K^+^ in the extracellular solutions (high [K^+^] solution with threefold increase of the KCl concentration to 6 mM and low K^+^ solution of 1 mM) were used to determine the ionic nature of the conductance. Anandamide (R&B Systems) was supplied pre-dissolved in anhydrous ethanol, 5mg/ml and stored in aliquots at -20°C. Anandamide was dissolved in physiological solution to reach 3 μM concentration and applied for 20 minutes. The TASK-1 blocker AVE1231 (kindly provided by Sanofi, Germany) was stored in 10 mM aliquots and dissolved in physiological solution to reach 10 μM and applied for at least 20 min.

### Electrophysiology

For the intracellular experiments, spinal neurons were recorded using patch electrodes pulled from borosilicate glass microcapillaries. Whole-cell recordings were performed in current- or voltage-clamp mode using a Multiclamp 700B amplifier (Molecular Devices). Bridge balance and pipette capacitance compensation were automatically adjusted. Patch electrodes had resistances of 5–10 M and contained the following (in mM): 105 K gluconate, 30 KCl, 10 Na phosphocreatine, 5 HEPES, 0.001 GTP and 0.003 ATP. The pH was adjusted to 7.4 with KOH and the osmolarity to 250 mOsm with H_2_O. These cells are all putative motoneurons. They were abundant in the gray matter and after the longitudinal transection their large soma become visible (Fig 1A2), due to their position and size and our previous experience, they were most certainly motoneurons [17]. They had an input resistance that varied from 40 to 160 M ohms at rest, which correlates to large size motoneurons. Hyperpolarizing steps where injected and the currents were monitored 10 and 20 minutes after pH change or drug application. Since the effects for substance P were not reversible within reasonable times for whole-cell recordings, only one neuron could be analyzed for each spinal cord preparation for all experiments with whole-cell recording. Input resistance measurements were done by measuring the currents induced by hyperpolarizing steps to -70 and -100 mV. The slope of the I-V relationship was calculated by the ratio of the current divided by the ratio of the voltage ((y_2_-y_1)_/(x_2_-x_1_)) between -70 and -100 mV. A higher slope value indicates a steeper incline and therefore a higher input resistance.

Data were acquired with Clampex software and analyzed using Clampfit (pCLAMP 10, Molecular Devices, CA, USA) and Spike2 4.16 software (Cambridge Electronic Design, Cambridge, UK). Summary statistics and the values shown in the figures are reported as standard error of the mean (± SEM) and “n” represents the number of experiments. The significance was determined using Student’s t-test with a 95% confidence interval.

## Results

### The resting potential and membrane currents are dependent on extracellular K^+^ concentrations

Background channels such as two-pore K^+^ channels allow ionic flux with an equilibrium potential that can be calculated by the Nernst equation. Since both the voltage and the concentration gradients influence the movement of ions, we characterized the channels by applying steps under whole-cell voltage clamp recordings in the presence of tetrodotoxin (TTX) and Kynurenic acid to block sodium channels and glutamate receptors. The result was a linear I-V relationship of membrane conductance over a wide range of command voltages with a reversal potential of mixed currents around -60 mV (Fig 1B1), suggesting that possibly low-voltage-activated calcium channels could also be involved [18, 19]. Furthermore, at different extracellular K^+^ concentrations the I-V relationship and the reversal potential shifted with a direction consistent, with a change in E_K_ (Fig 1B2). A three-fold increase in potassium concentration from 2.1 mM in control, caused a membrane depolarization (average 9.1 mV, ± 2.3, p < 0.05, n = 4) and in contrast, at low K^+^ concentrations (1mM) a membrane hyperpolarization was observed (average 6 mV, ± 1.9, p < 0.05, n = 4) (Fig 1C). Fig 1D shows the current fluctuations of a cell a cell at -70 mV and -100 mV at the different K^+^ concentrations. During a high K^+^ concentration (6 mM) more current was needed to hold the cell at hyperpolarized levels and at low K^+^ concentration (1 mM) a reduction in current amplitude was noticeable. In both cases the current amplitude was reversible to control conditions (2.1 mM). These results indicate that at hyperpolarized levels background K^+^ channels are active and contribute to the change in conductance.

### Substance P depolarizes the membrane and increases the input resistance

**Fig 2:**
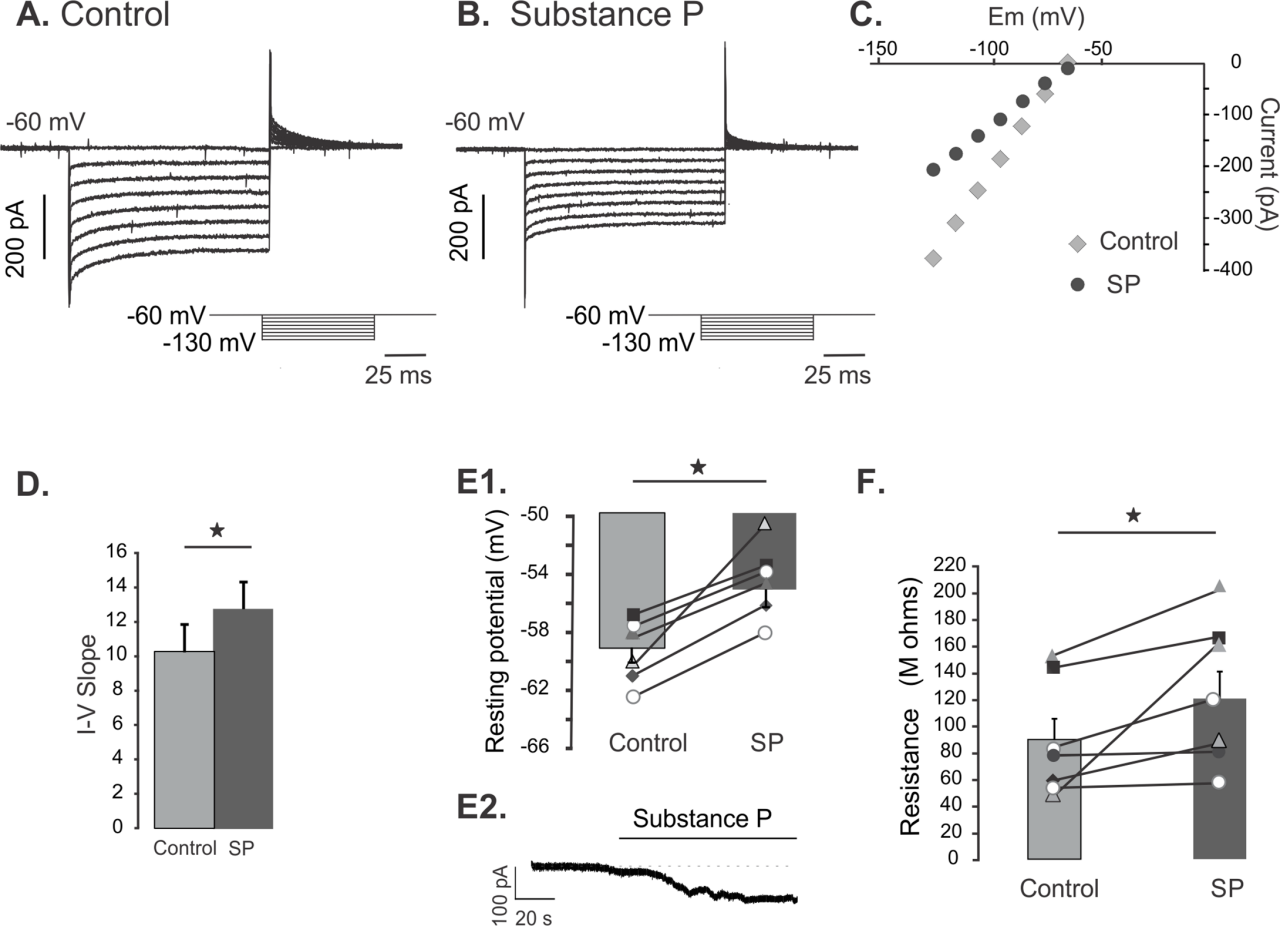
Substance P inhibits K^+^ background conductance. A. Current traces from hyperpolarizing voltage steps (-60 to -130 mV) applied in control conditions. B. Current traces from hyperpolarizing voltage steps applied to the same cell as in A during substance P bath application. C. The current-voltage relation (I-V) of the traces shown in A and B revealing a decrease in conductance with substance P (SP = substance P). D. Change of the I-V slope between control conditions and substance P application (n = 7 single star = p < 0.05). E. Resting membrane potentials recorded from cells in control and after substance P (1 μm) application. Bars show the average membrane potential in control and during substance P application. No current compensation was made in any case (n = 7, single star = p < 0.05). F. Input resistance in control and after substance P application computed from IV measurements under voltage clamp (n = 7, single star = p < 0.05). Bars show the average resistance in control and during substance P application.

To study the effect of substance P on background K^+^ channels, we recorded the responses in voltage clamp and current clamp. Hyperpolarizing steps were measured under control conditions (Fig 2A) and during substance P bath application (Fig 2B). The I-V relationship during control conditions and during substance P application shows a reduction of inward current (Fig 2C and 2D). During current clamp, all cells responded with a depolarization during substance P application. Fig 2E shows resting membrane potential in control conditions and during substance P application (+4 mV, ± 1.4 mV, p = 0.05, n = 7). Consistently, substance P caused an increase in input resistance of all cells tested (42%, ± 24%, p = 0.05, n = 7). The magnitude of the response varied between cells, this variation is likely to be related to the cell size. Fig 2F shows the input resistance before and after substance P application.

Taken together, the results suggest that the depolarization and the increase in input resistance are both driven by inhibition of a K^+^ conductance.

### Response to extracellular changes of pH

**Fig 3:**
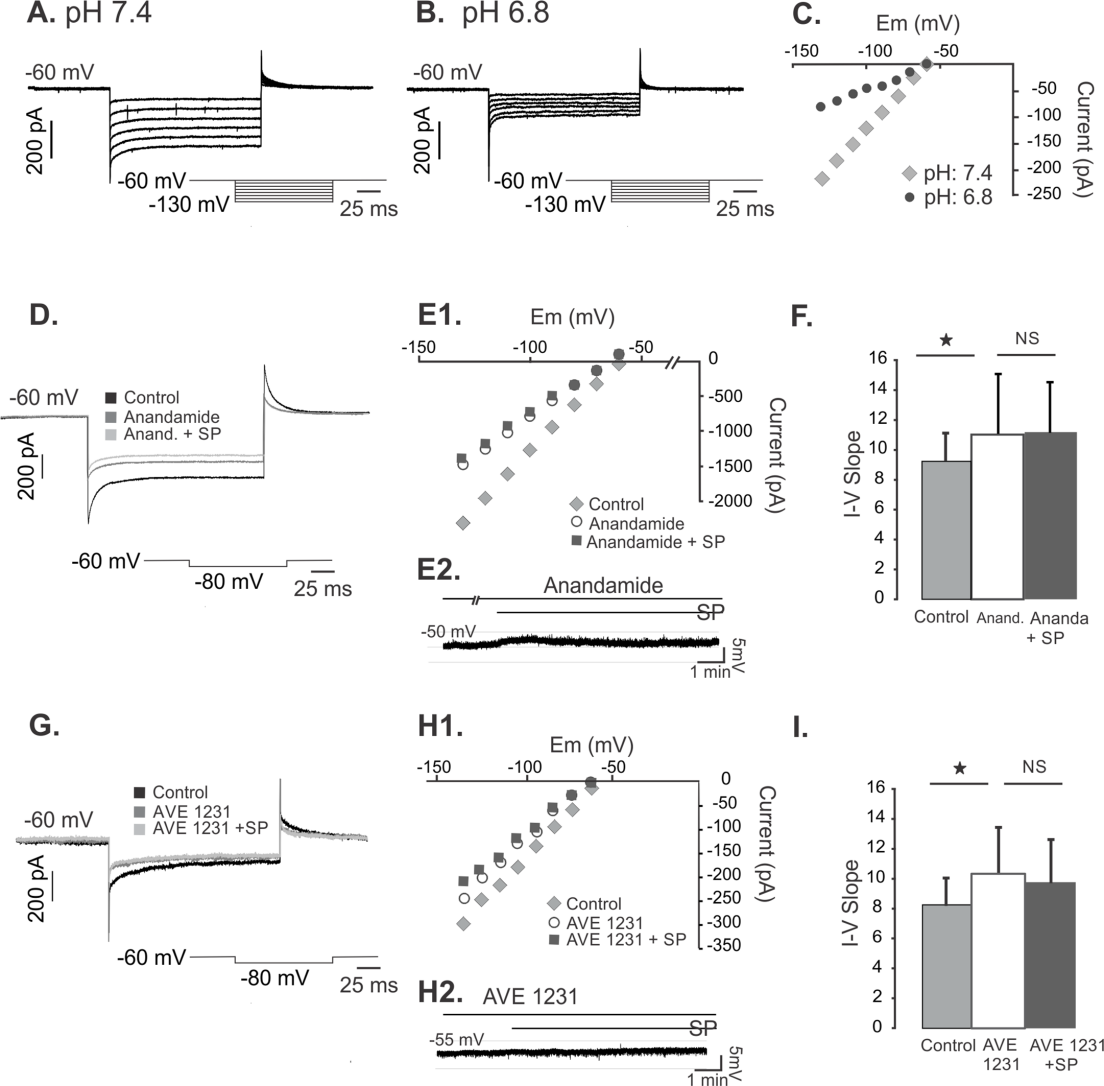
Background K^+^ conductance reduced by low pH, anandamide and AVE1231 suggesting an involvement of the TASK-1 K^+^ channel. A. Current traces from hyperpolarizing voltage steps in control conditions (pH 7.4). Inset: voltage step protocol. B. Current traces from hyperpolarizing voltage steps applied to the same cell as in A during acidification (pH 6.8) of the extracellular medium. C. The current-voltage relation (I-V) of the traces shown in A and B revealing a decrease in conductance at pH 6.8. D. Current traces from a hyperpolarizing voltage step (-80 mV) in control conditions, during anandamide alone and with anandamide and substance P. E1. The current-voltage relation (I-V) of the traces shown in D revealing a decrease in conductance with anandamide and a diminished effect of substance P in the presence of anandamide. E2. Current clamp trace during anandamide preincubation where substance P depolarizing effect is partially blocked. F. Change of the I-V slope between control conditions and anandamide (n = 7) and no further change of the slope after addition of substance P in the presence of anandamide. G. Current traces from a hyperpolarizing voltage step (-80 mV) in control conditions, during AVE1231 alone and with AVE1231 and substance P. H1. The current-voltage relation (I-V) of the traces shown in G revealing a decrease conductance with AVE1231, and diminished effect of substance P in the presence of AVE1231. H2. Current clamp trace during AVE 1231 where substance P depolarizing effect is completely blocked. I. Change of the I-V slope between control conditions and AVE1231 (n = 5) and no further change of the slope after addition of substance P in the presence of AVE1231 and substance P (n = 5).

A subtype of background channels, TASK-1, are known to be sensitive to pH. To determine if changes in the K^+^ conductance, we tested the current response to hyperpolarizing steps under control conditions (pH: 7.4, Fig 3A) and in lower extracellular pH (pH: 6.8, Fig 3B). Acidification induced a significant reduction of the response to current steps and a shift in the voltage-current relationship (Fig 3C). (Measured at -70 mV: 40%, ± 8%, p = 0.05, n = 4). As only TASK-1 K^+^ channels are inhibited by acidification [12, 20], the linear I-V relationship together with the pH sensitivity support the presence of this type of channel in lamprey neurons, and suggests that they are partially responsible for the background K^+^ conductance.

### Selective blockade of TASK-1 type K^+^ Channels

Two-pore domain K^+^ channels such as TASK-1 show a relative insensitivity to classical K^+^ channel blockers as TEA or Cs^+^ [21, 22]. However are blocked directly by anandamide, an cannabinoid receptor 1 agonist (CB1) [23]. It has been suggested that at low concentrations, anandamide has effects independent of the CB_1_ receptors [24]. To determine whether substance P inhibits TASK-1 K^+^ currents, we treated the cells with bath applied anandamide to block these channels. Anandamide indeed reduced the conductance during hyperpolarizing steps. Fig 3D shows an anandamide induced current reduction for a -80 mV step from -60mV followed by the response during anandamide plus substance P. Fig 3E1 shows the I-V curve during control conditions, anandamide alone and in the presence of substance P. Following the inhibition of the K^+^ conductance by anandamide, substance P induced practically no change in the holding current. In current clamp conditions, anandamide blocks the depolarization of substance P (Fig 3E2). However in 3 of 6 cases, a modest remaining depolarization was noticed, suggesting that substance P might also weakly activate a cation channel in some motoneurons [25]. Fig 3F shows a significant change of the slope between control conditions and anandamide application (p < 0.05, n = 7) however; no further reduction was seen during substance P application (n = 4).

To provide further evidence of the background conductance inhibited by substance P we applied the TASK-1 blocker AVE1231 [2]. AVE1231 inhibited background conductance and moreover, prevented the substance P effect. Fig 3G shows traces during control conditions and that AVE1231 induced a current reduction at -80 mV. No further reduction of the conductance was seen with substance P in the presence of the blocker, and neither a depolarization under current clamp conditions (n = 3 of 3). Fig 3H1 shows the I-V curve during control conditions, AVE1231 alone and in the presence of substance P. Fig 3H2 shows a representative trace of a block of depolarization in current clamp conditions. Fig 3I shows a significant change of the slope between control conditions and AVE1231 application (p < 0.05, 5 of 5) however, no further reduction was observed during the substance P application (n = 5). These data provide further support that substance P acts on K+ channels of the TASK-1 channel type.

## Discussion

### The resting potential and membrane depolarization

Background selective K^+^ channels are a primary source for the current occurring at resting membrane potential and therefore give rise to a negative membrane potential [25].

When the concentration of K^+^ outside the neuron is altered, the slope of the I-V relationship does not change; however, there is a shift in the membrane potential. This suggests that the background current is carried out by K^+^.

### Substance P depolarizes the membrane and increases the input resistance

Substance P reduced the slope in the I-V relation at negative potentials, which means there was an increase in resistance. The physiological correlate of an increase in input resistance is a closure of ion channels. The fact that we also observed a depolarization with substance P under current clamp, means that the ion channels that are affected have a reversal potential negative to the resting potential and are therefore most likely background K^+^ channels.

G-protein coupled receptors have been reported to inhibit K^+^ currents in mammals and substance P has been shown to inhibit background K^+^ channels of the two-pore, TASK-1 subtype [3].

Sodium leak channels (NALCN) although not completely selective can be activated by substance P [26] [27], and an activation would results in a decrease of the input resistance. In our case, however, substance P instead causes an increase in input resistance. NALCN channel thus not contribute to the substance P effects observed here.

### Background K^+^ channel characterization

pH changes in the central nervous system is implicated in a number of physiological processes. Under physiological conditions the pH is around 7.4. Lowering the pH causes TASK-1 Two-pore domain K^+^ channels to close, providing a tool for its characterization [12]. At a pH of 6.8 we had a significant response, very similar to the one with substance P, suggesting that substance P would act via TASK-1 channels.

The possible involvement of the two-pore domain K^+^ channel type was also tested by applying anandamide. Anandamide is a CB_1_ agonist that has been shown to have multiple effects and to directly block TASK-1 type two-pore K^+^ channel at low concentrations [23]. In the presence of the anandamide, the slope of the I-V curve was shifted, indicating an increase of the input resistance consistent with an inhibition of a K^+^ current. Furthermore, substance P did not have an effect in its presence presumably due to previous blockade of the TASK-1 by anandamide, providing further evidence that two-pore, TASK-1 is the type of background K^+^ channel modulated by substance P. Anandamide was also able to partially block the effect on the depolarization caused by substance P. The remaining effect observed only in some cases, might be associated with a cation channel activation and possibly differences between fast and slow motoneurons.

The blocker AVE1231 is a novel compound reported to be potent and specific TASK-1 blocker in humans and rodents [2, 28]. Motoneurons are known to express TASK-1 and TASK-3 in rodents [16]. AVE1231 caused a significant reduction of the background K^+^ current compared to control, however, to a lesser extent than anandamide and in current clamp conditions, it is also able to block the depolarization of substance P.

**Fig 4:**
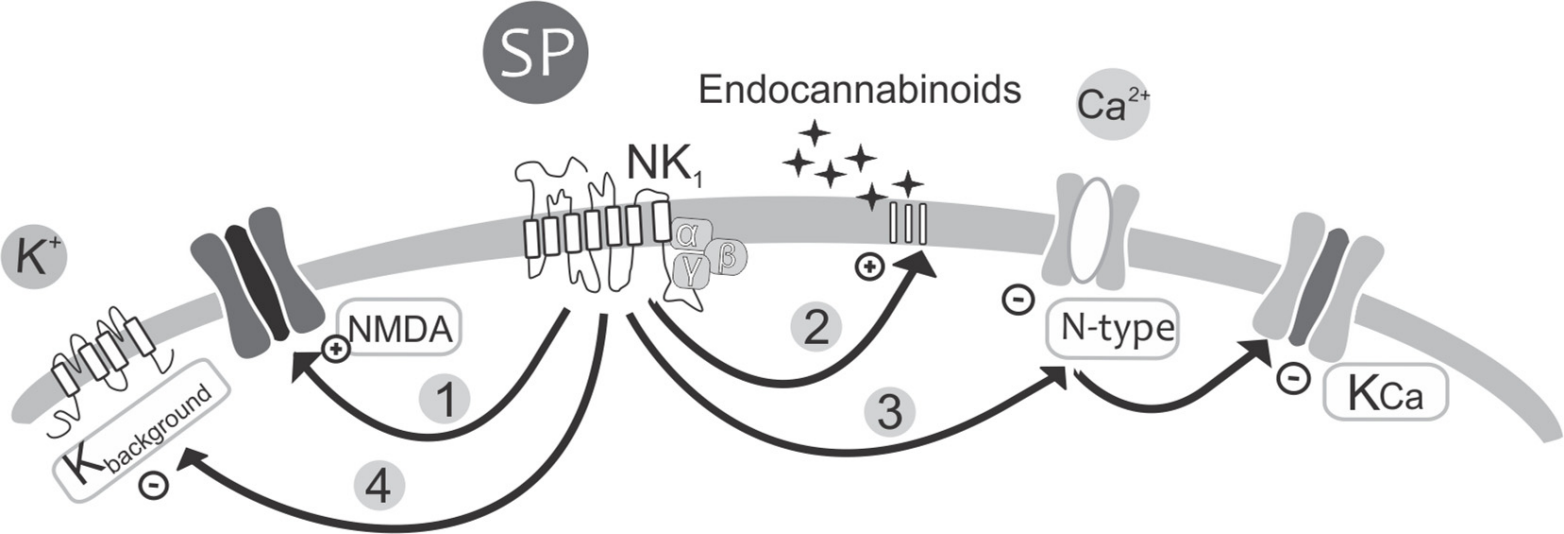
Substance P activation of NK_1_ receptors modulates several signaling pathways. 1. Endogenous release of substance P, activates NK_**1**_ receptors which via Protein kinase C (PKC) potentiate NMDA receptors (Parker and Grillner 1999). Substance P increases the frequency of the NMDA-induce oscillations in the presence of TTX, an effect that likely contributes to the substance P increase of the locomotor frequency. 2. Endocannabinoids: In response to NK_**1**_ activation by substance P, endocannabinoids can either be synthesized from DAG or released by Ca^2+^ from internal stores. The released endocannabinoids act as retrograde messengers to depress inhibitory synaptic transmission via presynaptic receptors (CB_**1**_), thereby reducing glycinergic inhibition. 3. K_**Ca**_: Substance P application causes a reduction in calcium currents in motoneurons and commissural interneurons, primarily by inhibiting N-type (Ca_**v**_2.2) Ca^2+^ channels, which in turn will affect the activation of Ca^2+^ dependent channels K_**Ca**_. 4. K_**background**_: Inhibition of background channels by substance P induces membrane depolarization, increased membrane resistance and consequently increases the firing rate, which could have great impact at the cellular level as well as during fictive locomotion.

Two-pore domain channels have been found in most examined tissues [29]. In mammals TASK-1 channels have been found in the central nervous system [30]. Closure of these channels by neuromodulators would have profound effects on neuronal networks. A large scale effect from depolarization and a higher input resistance would occur that would increase excitability. This may, then, be a mechanism contributing to the increased burst frequency caused by substance P in the lamprey locomotor network (summarized in Fig 4). As the rhythm is affected by substance P it is likely that this effects are exerted at the interneuron level in the CPG.

## References

[1] Nicoll RA, Malenka RC, Kauer JA. Functional comparison of neurotransmitter receptor subtypes in mammalian central nervous system. Physiol Rev, 1990 70(2): p. 513–65. 1690904 10.1152/physrev.1990.70.2.513

[2] Ehrlich JR, Ocholla H, Ziemek D, Rütten H, Hohnloser SH, Gögelein H. Characterization of human cardiac Kv1.5 inhibition by the novel atrial-selective antiarrhythmic compound AVE1231. J Cardiovasc Pharmacol, 2008 51(4): p. 380–7. 10.1097/FJC.0b013e3181669030 18427281

[3] Talley EM, Lei Q, Sirois JE, Bayliss DA. TASK-1, a two-pore domain K+ channel, is modulated by multiple neurotransmitters in motoneurons. Neuron, 2000 25(2): p. 399–410. 10719894 10.1016/s0896-6273(00)80903-4

[4] Koizumi H, Smerin SE, Yamanishi T, Moorjani BR, Zhang R, Smith JC. TASK channels contribute to the K+-dominated leak current regulating respiratory rhythm generation in vitro. J Neurosci, 2010 30(12): p. 4273–84. 10.1523/JNEUROSCI.4017-09.2010 20335463 PMC2950010

[5] Grillner S. The motor infrastructure: from ion channels to neuronal networks. Nat Rev Neurosci, 2003 4(7): p. 573–86. 12838332 10.1038/nrn1137

[6] Barthe JY, Clarac F. Modulation of the spinal network for locomotion by substance P in the neonatal rat. Exp Brain Res, 1997 115(3): p. 485–92. 9262203 10.1007/pl00005718

[7] Thörn Pérez C, Hill RH, Grillner S. Endogenous tachykinin release contributes to the locomotor activity in lamprey. J Neurophysiol, 2007 97(5): p. 3331–9. 17360825 10.1152/jn.01302.2006

[8] Parker D, Grillner S. Cellular and synaptic modulation underlying substance P-mediated plasticity of the lamprey locomotor network. J Neurosci, 1998 18(19): p. 8095–110. 9742176 10.1523/JNEUROSCI.18-19-08095.1998 PMC6793019

[9] Parker D, Grillner S. Activity-dependent metaplasticity of inhibitory and excitatory synaptic transmission in the lamprey spinal cord locomotor network. J Neurosci, 1999 19: p. 1647–1656. 10024351 10.1523/JNEUROSCI.19-05-01647.1999 PMC6782186

[10] Thörn Pérez C, Hill RH, El Manira A, Grillner S. Endocannabinoids mediate tachykinin-induced effects in the lamprey locomotor network. J Neurophysiol, 2009 102(3): p. 1358–65. 10.1152/jn.00294.2009 19571197

[11] Schneider ER, Anderson EO, Gracheva EO, Bagriantsev SN. Temperature Sensitivity of Two-Pore (K2P) Potassium Channels. Curr Top Membr, 2014 74: p. 113–33. 10.1016/B978-0-12-800181-3.00005-1 25366235 PMC4794111

[12] Duprat F, Lesage F, Fink M, Reyes R, Heurteaux C, Lazdunski M. TASK, a human background K+ channel to sense external pH variations near physiological pH. Embo J, 1997 16(17): p. 5464–71. 9312005 10.1093/emboj/16.17.5464 PMC1170177

[13] Sirois JE, Lei Q, Talley EM, Lynch C 3rd, Bayliss DA. The TASK-1 two-pore domain K+ channel is a molecular substrate for neuronal effects of inhalation anesthetics. J Neurosci, 2000 20(17): p. 6347–54. 10964940 10.1523/JNEUROSCI.20-17-06347.2000 PMC6772985

[14] Maggi CA, Schwartz TW. The dual nature of the tachykinin NK1 receptor. Trends Pharmacol Sci, 1997 18(10): p. 351–5. 9357319 10.1016/s0165-6147(97)01107-3

[15] Kettunen P, Hess D, El Manira A. mGluR1, but not mGluR5, mediates depolarization of spinal cord neurons by blocking a leak current. J Neurophysiol, 2003 90(4): p. 2341–8. 12815014 10.1152/jn.01132.2002

[16] Perrier JF, Rasmussen HB, Christensen RK, Petersen AV. Modulation of the intrinsic properties of motoneurons by serotonin. Curr Pharm Des, 2013 19(24): p. 4371–84. 23360270 10.2174/13816128113199990341

[17] Thörn Pérez C, Hill HR, Grillner S. Substance P reduces calcium influx trough N-type but not L-type calcium channels in lamprey spinal neurons. in Program No. 577.15. 2012.

[18] Tegnér J, Hellgren-Kotaleski J, Lansner A, Grillner S. Low-voltage-activated calcium channels in the lamprey locomotor network: simulation and experiment. J Neurophysiol, 1997 77(4): p. 1795–812. 9114237 10.1152/jn.1997.77.4.1795

[19] Wang D, Grillner S, Wallen P. Calcium dynamics during NMDA-induced membrane potential oscillations in lamprey spinal neurons—contribution of L-type calcium channels (CaV1.3). J Physiol, 2013 591(Pt 10): p. 2509–21. 10.1113/jphysiol.2012.248526 23440960 PMC3678040

[20] Leonoudakis D, Gray AT, Winegar BD, Kindler CH, Harada M, Taylor DM, et al An open rectifier potassium channel with two pore domains in tandem cloned from rat cerebellum. J Neurosci, 1998 18(3): p. 868–77. 9437008 10.1523/JNEUROSCI.18-03-00868.1998 PMC6792778

[21] Koh DS, Jonas P, Bräu ME, Vogel W. A TEA-insensitive flickering potassium channel active around the resting potential in myelinated nerve. J Membr Biol, 1992 130(2): p. 149–62. 1291683 10.1007/BF00231893

[22] Lopes CM, Gallagher PG, Buck ME, Butler MH, Goldstein SA. Proton block and voltage gating are potassium-dependent in the cardiac leak channel Kcnk3. J Biol Chem, 2000 275(22): p. 16969–78. 10748056 10.1074/jbc.M001948200

[23] Maingret F, Patel AJ, Lazdunski M, Honoré E. The endocannabinoid anandamide is a direct and selective blocker of the background K(+) channel TASK-1. Embo J, 2001 20(1–2): p. 47–54. 11226154 10.1093/emboj/20.1.47 PMC140203

[24] Zygmunt PM, Petersson J, Andersson DA, Chuang H, Sørgård M, Di Marzo V, et al Vanilloid receptors on sensory nerves mediate the vasodilator action of anandamide. Nature, 1999 400(6743): p. 452–7. 10440374 10.1038/22761

[25] Hille B. Ion Channels of Excitable Membranes. Third ed. 2001, Sunderland, MA: Sinauer Associates, Inc. 813.

[26] Lu B, Su Y, Das S, Wang H, Wang Y, Liu J, et al Peptide neurotransmitters activate a cation channel complex of NALCN and UNC-80. Nature, 2009 457(7230): p. 741–4. 10.1038/nature07579 19092807 PMC2810458

[27] Ren D. Sodium leak channels in neuronal excitability and rhythmic behaviors. Neuron, 2011 72(6): p. 899–911. 10.1016/j.neuron.2011.12.007 22196327 PMC3247702

[28] Schiekel J, Lindner M, Hetzel A, Wemhöner K, Renigunta V, Schlichthörl G, et al The inhibition of the potassium channel TASK-1 in rat cardiac muscle by endothelin-1 is mediated by phospholipase C. Cardiovasc Res, 2013 97(1): p. 97–105. 10.1093/cvr/cvs285 22977011

[29] Lesage F. Pharmacology of neuronal background potassium channels. Neuropharmacology, 2003 44(1): p. 1–7. 12559116 10.1016/s0028-3908(02)00339-8

[30] Medhurst AD, Rennie G, Chapman CG, Meadows H, Duckworth MD, Kelsell RE, et al Distribution analysis of human two pore domain potassium channels in tissues of the central nervous system and periphery. Brain Res Mol Brain Res, 2001 86(1–2): p. 101–14. 11165377 10.1016/s0169-328x(00)00263-1

